# Environmentally Enriched Male Mink Gain More Copulations than Stereotypic, Barren-Reared Competitors

**DOI:** 10.1371/journal.pone.0080494

**Published:** 2013-11-25

**Authors:** María Díez-León, Jeff Bowman, Steve Bursian, Hélène Filion, David Galicia, Jeannette Kanefsky, Angelo Napolitano, Rupert Palme, Albrecht Schulte-Hostedde, Kim Scribner, Georgia Mason

**Affiliations:** 1 Department of Animal and Poultry Science, University of Guelph, Guelph, Ontario, Canada; 2 Ontario Ministry of Natural Resources, Wildlife Research and Development Section, Trent University, Peterborough, Ontario, Canada; 3 Department of Animal Science, Michigan State University, East Lansing, Michigan, United States of America; 4 Department of Biology Laurentian University, Sudbury, Ontario, Canada; 5 Department of Zoology and Ecology, University of Navarra, Pamplona, Spain; 6 Department of Fisheries and Wildlife, Michigan State University, East Lansing, Michigan, United States of America; 7 Department of Biomedical Sciences, University of Veterinary Medicine Vienna, Vienna, Austria; CNRS, France

## Abstract

Wild carnivores in zoos, conservation breeding centres, and farms commonly live in relatively small, unstimulating enclosures. Under these captive conditions, in a range of species including giant pandas, black-footed ferrets, and European mink, male reproductive abilities are often poor. Such problems have long been hypothesized to be caused by these animals' housing conditions. We show for the first time that rearing under welfare-improving (i.e., highly valued and stress-reducing) environmental enrichments enhances male carnivores' copulatory performance: in mate choice competitions, enriched male American mink (*Neovison vison*) mated more often than non-enriched males. We screened for several potential mediators of this effect. First was physiological stress and its impact on reproductive physiology; second, stress-mediated changes in morphology and variables related to immunocompetence that could influence male attractiveness; and third, behavioural changes likely to affect social competence, particularly autistic-like excessive routine and repetition (‘perseveration’) as is reflected in the stereotypies common in captive animals. Consistent with physiological stress, excreted steroid metabolites revealed that non-enriched males had higher cortisol levels and lower androgen levels than enriched conspecifics. Their *os penises* (bacula) also tended to be less developed. Consistent with reduced attractiveness, non-enriched males were lighter, with comparatively small spleens and a trend to greater fluctuating asymmetry. Consistent with impaired social competence, non-enriched males performed more stereotypic behaviour (e.g., pacing) in their home cages. Of all these effects, the only significant predictor of copulation number was stereotypy (a trend suggesting that low bodyweights may also be influential): highly stereotypic males gained the fewest copulations. The neurophysiological changes underlying stereotypy thus handicap males sexually. We hypothesise that such males are abnormally perseverative when interacting with females. Investigating similar problems in other taxa would be worthwhile, since many vertebrates, wild and domestic, live in conditions that cause stereotypic behaviour and/or impair neurological development.

## Introduction

Breeding problems are common in certain captive wild species, including several endangered Carnivora. To illustrate, in zoos and captive breeding centres many captive male European mink (*Mustela lutreola*: [1]), black-footed ferrets (*Mustela nigripes*: [2]), giant pandas (*Ailuropoda melanoleuca*: [3], [4]) and some felids [5], [6] fail to sire offspring; captive females may be acyclic (e.g. black-footed cat *Felis nigripes* and sand cat *Felis margarita*, [7]); and infant mortality can also be excessive (e.g. in black-footed ferrets [8], [9]; giant pandas [10]; African wild dogs *Lycaon pictus* [11]; and other carnivores [12]). Several authors have attributed this compromised reproductive performance to sub-optimal housing or husbandry [8], [12], [13], [14], [15], [16], [17], [18], [19]. For wild carnivores in zoos and breeding centres, the differences between natural and manmade environments can certainly be stark: the sizes of polar bear enclosures, for instance, have averaged approximately one millionth the area of even the smallest home ranges in the wild [20]. Furthermore, stereotypic behaviours (abnormal repetitive movements such as pacing, e.g. [21]), are common in this taxon under these conditions: in behavioural studies of captive wild carnivores, 82% of individuals displayed some type of stereotypic behaviour [22]. Caused by motivational frustration and/or changes in brain function [23], these typically indicate inappropriate husbandry [24]. To date, researchers attributing reproductive problems to wild animals' housing conditions have emphasised the likely adverse effects of stress on reproductive physiology. This idea is plausible because a number of studies, primarily of laboratory and farmed species, have shown that a range of aversive stimuli can act to reduce males' androgen levels, testis size, sperm production and effective spermatogenesis [25],[26],[27],[28], and also compromise females' receptivity, neonate birthweights, litter sizes, and lactation [29],[30,31],[32],[33],[34],[35]. These effects have been shown to reflect inhibition of the hypothalamic-pituitary-gonadal (HPG) endocrine axis by hypothalamic-pituitary-adrenal (HPA) hormones [36],[37]. Furthermore, some HPG-dependent variables can be compromised in impoverished housing conditions (e.g. reduced testis size in Siberian hamsters, *Phodopus sungorus*: [38]). Thus if captive conditions cause chronic HPA activation, reproductive problems might well ensue.

We further hypothesise two additional processes by which sub-optimal housing might lead to poor breeding in captive mammals. These ideas have been suggested respectively by behavioural ecology research on a wide range of species (including humans), and by behavioural neuroscience research (primarily on laboratory rodents). Our first novel hypothesis is that poor captive rearing environments act as “developmental stressors”, altering the ontogeny of young males in such a way as to diminish their attractiveness to females as adults. To illustrate, larger mates are often preferred by females than smaller mates [39],[40]; yet developmental stress can compromise growth rates [25],[41], and captive conditions are known to render some males smaller than their free-living conspecifics (e.g. in black-footed ferrets: [42],[43]; jaguars, *Panthera onca*: [6]). Morphological symmetry is another attribute that is generally preferred by females choosing mates [44],[45]. Deviations from symmetry (assessed as "fluctuating asymmetry", [46] cited in [47]) can be induced by stress during early ontogenic stages [48],[49], and symmetry is also potentially compromised by development in barren cages [50],[51]. Finally, immunocompetence and attributes that correlate with this are also important in mate choice [52], and yet impaired by both known stressors [53] and by impoverished housing [38],[54],[55]. Males raised in barren environments therefore seem at risk of developing into physically unattractive adults, but this idea has never been experimentally tested. Our second novel hypothesis is that poor developmental environments impair brain function, so as to render courtship, mating and/or parental behaviours less effective. In male songbirds, to illustrate, the neural substrates for attractive singing require low stress rearing conditions [49],[56], while in mammals, barren rearing environments have two well-studied effects on brain and behaviour that are likely to compromise interactions with potential mates and/or dependent offspring. First, barren environments retard hippocampal and cerebral development (and even overall brain weight [57],[58]), causing reduced learning abilities (e.g. [57],[59]): potentially important because learning abilities increase attractiveness in several species [60],[61] and also help improve young adults' abilities to copulate as well as to care for their offspring [62],[63]. Second, barren environments induce stereotypic behaviours (e.g. [24]), in part by causing basal ganglia dysfunction (e.g. [64]). Bouts of stereotypic activity may be problematic if they interrupt normal intra-specific interactions ([65]; see [66] for a zoo example). Furthermore, stereotypic behaviour typically reflects generalised changes in behavioural control that are manifest as abnormal tendencies to repeat actions and over-utilise fixed routines (‘perseveration’). Such impairments in behavioural organisation characterise human disorders featuring stereotypic behaviour [64],[67],[68], and make affected subjects poor at the flexible ‘give and take’ of normal social interaction. These impairments are also widespread in stereotypic captive animals (e.g. [23],[69],[70],[71]): perseveration has been found to be associated with stereotypic behaviour in all 15 species tested to date, with unknown implications for such animals' social, sexual and parental behaviours.

Adding environmental enrichment, i.e. welfare-enhancing stimuli to captive housing (e.g. [72]), has therefore been proposed as a solution to wild animals' breeding difficulties [1],,[3],[13],[15],[73]. There are several recent indications that enrichment is a useful approach. Enrichment enhances male mating success in laboratory-reared flies [74],[75]; prolongs reproductive lifespan in domestic hens [76]; and in mammals, enrichment seems to increase the number of infants succesfully weaned by females in laboratory mice [77] (though cf. [78]) and farmed mink [79]. However, no study has investigated the effects of enrichment in both sexes, through all stages of reproduction from mating inspections to weaning; nor has any study identified the mechanisms underlying any observed success. We therefore assessed whether raising captive Carnivora with enrichments improves their reproductive performance. We assessed our subjects from pre-copulatory phases (e.g. mate inspection) through to whelping, and also investigated the mediators of any effects. American mink (*Neovison vison*) were ideal model Carnivores because the enrichments that are beneficial for their welfare are well understood (e.g. [80],[81]); female behaviour and physiology suggest mate choosiness [82]; their stereotypies are known to be accompanied by increased perseveration [71],[83]; and small enclosures are commonly used for breeding endangered mustelids [2],[15],[84]).

If barren rearing and housing conditions impair carnivore reproduction, then enriched-reared mink (henceforth simply ‘enriched’) should mate more and/or produce more offspring. If such effects are mediated by HPA activation, then enrichment should reduce faecal glucocorticoid metabolites [85], and/or adrenal gland weight [55]) and have beneficial effects on traits relating to the HPG axis, with these effects correlating with reproductive benefits. If barren environments reduce male attractiveness, then enriched males should be heavier, more symmetrical, and/or show evidence of improved immunocompetence (e.g. smaller lymphoid organs such as thymus and spleen, [53],[86], both essential for cell-mediated immunity), with such changes in turn predicting increased mating success. Finally, if barren environments affect psychological traits important in courtship and/or maternal care, then enrichment should boost relative brain weight and/or reduce stereotypic behaviours, with these changes in turn predicting enhanced reproductive abilities.

## Methods

### Ethics statement

All research procedures were approved by both the University of Guelph Animal Care Committee (AUP #07R033) and the Michigan State Institutional Animal Care and Use Committee (AUF #04/07-041-00).

### Animals, housing, and differential rearing

**Figure 1:**
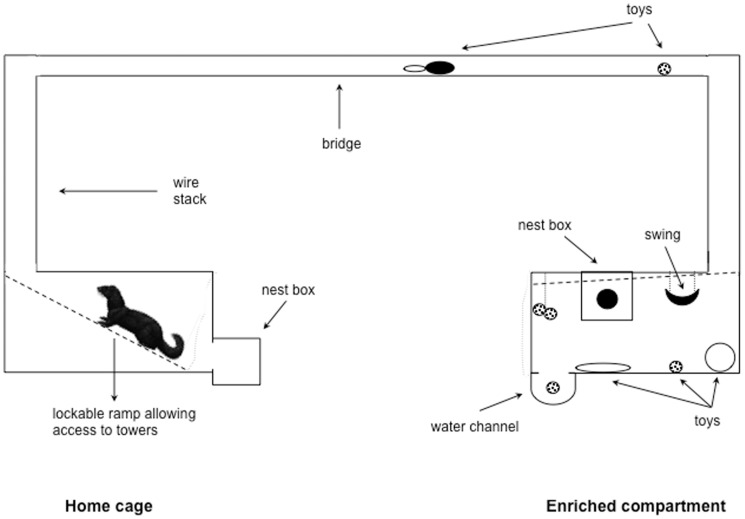
Diagram of the enriched treatment condition, showing the home cage and the connected enriched compartment. Two movable ramps could be raised (as shown in the enriched compartment in the figure) or lowered (as shown in the home cage in the figure) by a chain mechanism, in order to control access to the enriched compartment.

Sixty-four multiparous pregnant mink of the Black colourtype born at the Michigan State University's research farm were moved into an indoor facility where they were housed in wire mesh home cages (61×76×46cm) with solid plastic sidewalls and an attached plastic nest box (31×25×25cm, bedded all year round). They were provided with *ad libitum* water via a nipple drinker and approximately *ad libitum* food once daily. Non-enriched (NE) mink (N = 32) lived in unmodified versions of these, which resembled the smallest, least enriched enclosures that are used for breeding endangered mustelids [2],[15],[84]). Every alternate cage was an enriched (E) cage. Each of these E cages was supplied with a wire mesh vertical stack (56×15×121 cm) that each of the 32 E mink could access via lockable exit ramps (see e.g. [71], also Fig. 1). This vertical stack contained sloping ramps for the mink to climb, and led to a 13×244×13 cm overhead tunnel. The tunnel was connected to an identical stack at the other end, which mink could descend via sloping ramps to access a separate enriched compartment double the size of the home cage and provided with an extra empty wooden nest box (24×22×28 cm); novel objects (e.g. plastic toys) that were replaced with new items every month (highly preferred by mink [80]); a water channel (120 cm long) containing filtered circulating water for wading (mink are semi-aquatic [87] and highly motivated to access water baths [80]); and a variety of manipulable items (e.g. balls, chewable pet toys) plus tunnel/shelf-like structures (plastic pipes; a suspended, rigid plastic hammock) together known to reduce stereotypic behaviour and corticosteroid hormone output [81]. E mink thus had more space, more exercise opportunities, more sensory stimulation, and more access to reinforcement than NE mink. Parallel studies confirmed that these enriched compartments reduced stereotypic behaviour and corticosteroid outputs [71],[88],[89], and that mink were highly motivated to use them [89].

When infants (kits) were born, access to the E compartments was prevented until the young animals had open eyes and reasonable mobility (at approximately five weeks). Half the E and half the NE cages were designated ‘male’ and the other half ‘female’, such that each female subject would be raised between two males, one E and one NE. In each ‘male’ litter, one random male infant was selected as our subject; a female was similarly selected in each ‘female’ litter. Each subject's family was removed when infants were 52+/− 3 days old; it was then given an unrelated same age, same treatment, opposite sex companion until seven months of age (early adulthood). This ensured that all of our subjects were well-socialised and identically raised in terms of access to opposite-sex conspecifics. At seven months, companions were removed (adults being naturally solitary [87]). In total, our subject males (16 E, 16 NE) were differentially housed for 23 months, spanning two annual breeding seasons (after which they were killed). Thirty-two females were equally divided into ‘Year 1’ and ‘Year 2’ choosers. Year 1 choosers (8E, 8NE) were exposed to these differentially-housed males as potential mates in their first breeding season, when they were 10 months old; and differentially housed until being killed at 15 months (after weaning). Year 2 choosers (8E, 8NE) were mated with non-experimental males in Year 1; exposed to the differentially-housed males as potential mates in Year 2 when 22 months old; and differentially housed until being killed at 28 months (after weaning their second litters).

### Mating trials, and assessment of reproductive outcomes

**Figure 2:**
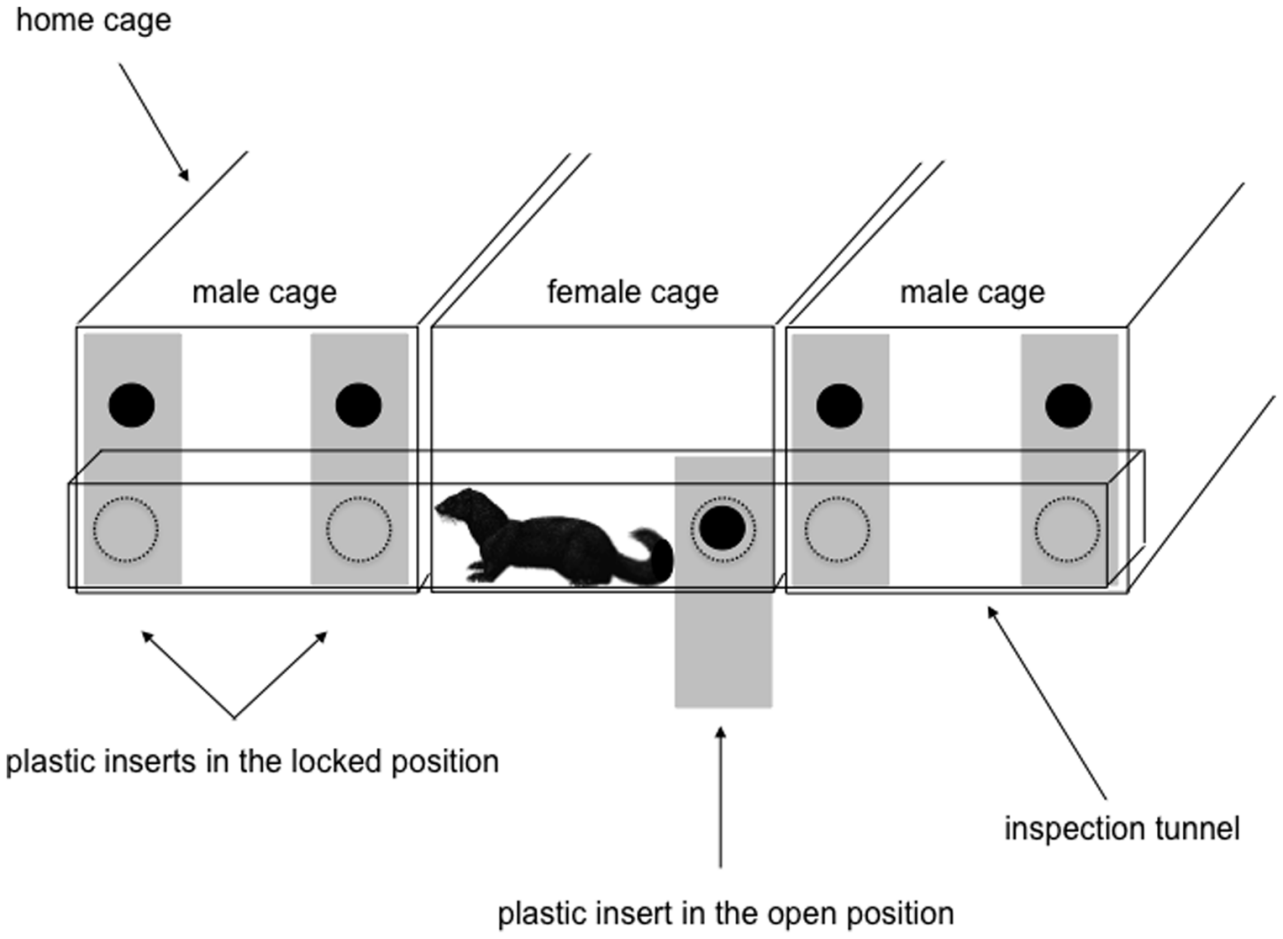
Diagram of the mate choice apparatus.

Each female was exposed to one pair of males (one E, one NE); each male pair was exposed to two chooser females (one in Year 1, one in Year 2). The mating trials utilised an apparatus that allowed each female to visit her two potential mates freely. This apparatus comprised of a tunnel connecting her cage with her two male neighbours, with five openings: one allowing her to exit/enter her own cage, and two per male cage to reduce males' abilities to prevent her leaving (Fig. 2). These openings were designed to exploit this species' great sexual dimorphism (females have approximately half the body size of males [82],[87]), being custom cut so that only the females could pass through them. Between trials these openings were blocked with solid inserts; while during trials, these were retracted to reveal the custom-sized holes allowing free passage of each female (Fig. 2). Before trials commenced, each female was given five days of access to familiarize with her apparatus, and males were removed for brief periods (<5 minutes, once or twice a day) to allow her to explore their cages. To ensure both males' cages would appear similar to females during mate choice trials, vertical stacks with ramps were attached to NE cages (but locked and inaccessible), and any enrichments in E males' homecages were removed. During mate choice trials, each female was moved to a new cage so that her potential mates were equally unfamiliar rather than longterm neighbours, to avoid differential exposure acting as confounds (e.g. each NE female might be less familiar with her E neighbour than her NE one, because E males spent time away in their E compartments; see [90] for the potential importance of familiarity). During trials, each E male was also locked out of his E compartment. Between trials, each female was returned to her own cage. The trials, each four hours long, were conducted daily for 21 consecutive days. During each trial, mink were scanned every 10 minutes to record visits by female to male; copulations (identified by the male holding the female in a position allowing intromission, the female's tail perpendicular to her body [82]); and stereotypic behaviours. Escalated aggression, defined (subjectively, based on the observer's prior experience with fighting mink) as agonistic interactions involving prolonged, repeated high-pitched screams, was used as a cue to terminate a trial; however, only two matings ever needed splitting because of these behaviours.

Parturition occurred approximately two months after mating. Kits were counted and weighed on post-natal day (PND) 1; plans to follow them for longer were abandoned, however, due to small sample sizes (see Results). They were pelted at nine months, and muscle samples collected to assess paternity (any dying earlier were similarly sampled; thus paternity data came from all kits). The PCR amplification conditions and quality control procedures for the paternity tests are described in [91]. In brief, 10 loci were used: Ma-2, Ma-19, Gg-7 [92], Mvis02, Mvis72, Mvis75, Mvis99, Mer09, Mer22, and Mer41 [93]. Allele counts, allele frequencies, expected and observed heterozygosities, and parentage analyses were calculated using CERVUS version 3.0 [94]. Each infant was assigned to one of his two possible fathers (blind to their treatment) based on complete exclusion or 70% or higher confidence. To help validate our behavioural data on mating (which were not collected blind to treatment), the behavioural data were compared to paternity data (see Results), since paternity analyses were conduced blind to treatment.

### Additional data collection from live subjects

Stereotypies were assessed over two 8-day periods (when mink first show these behaviours consistently, at 8 months old [95],[96], and then again at the end of our experiment, when the animals were 23 months old) (following methods in [71],[88],[89]). These were typically locomotor, involving whole-body movements like pacing or ‘nodding’ with the upper body, repeated at least three times consecutively [95]. Inactivity was scored so that stereotypies could be corrected for activity levels, to yield the measure that best correlates with perseveration (cf. e.g. [83],[97]).

After the second breeding season, faeces were collected and pooled over five consecutive days; stored in a −20°C freezer; then homogenized and processed [98]. Extracts were assayed (blind to treatment) for cortisol metabolite concentrations using a mink-validated enzyme immunoassay (EIA) for 11ß-hydroxyaetiocholanolone [99]. We also measured androgen metabolites with a testosterone (T) and epiandrosterone (EPI) EIA: these assays measure 17β-hydroxy- and 17-oxo-androgens, respectively [100],[101]. Since neither EIA is validated specifically for mink, and cross-reactivity with other steroids of adrenal origin is likely (indeed evident in our samples), we used glucocorticoid metabolite concentrations as covariates in all relevant analyses.

### *Post mortem* data collection

**Figure 3:**
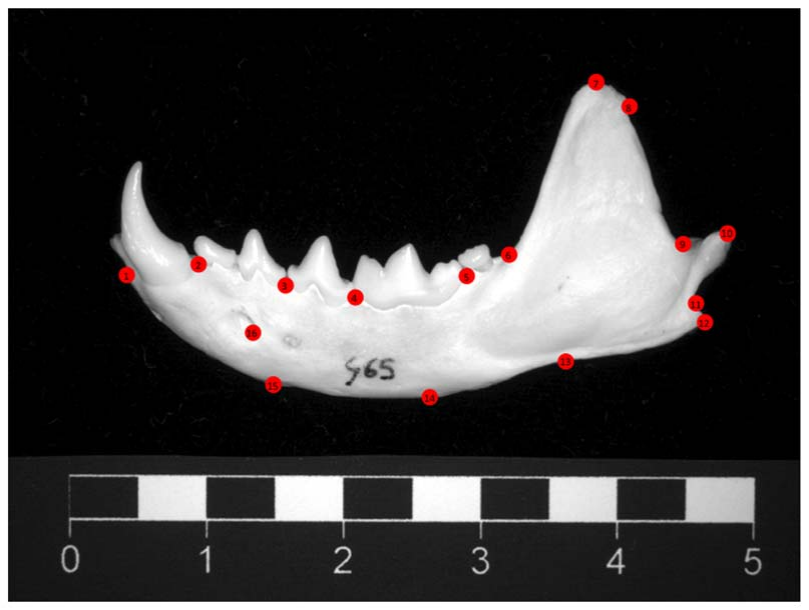
Location of landmarks on the labial side of the mandible.

**Figure 4:**
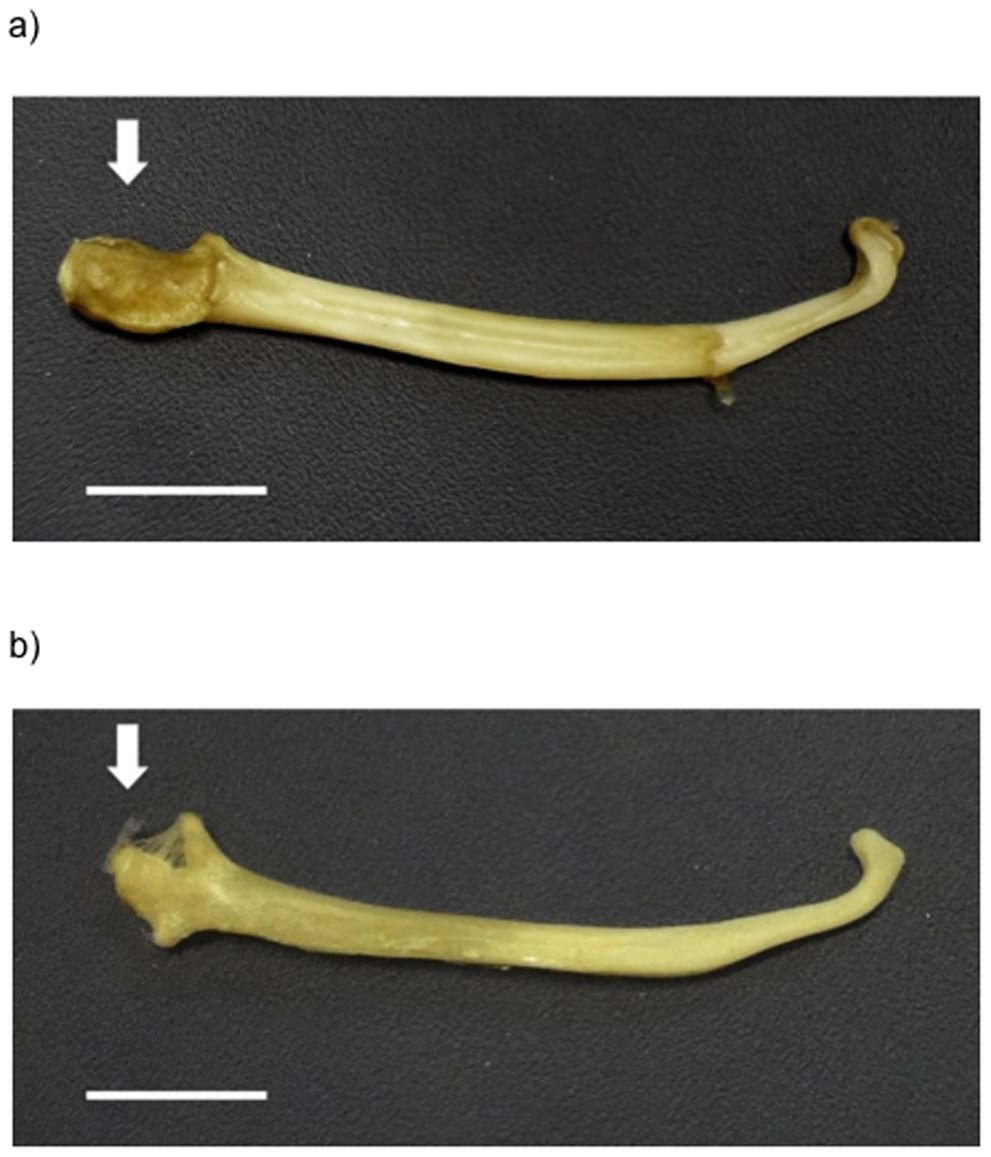
Mink bacula. a) baculum with a well-developed proximal process (mean score  = 30.2), and b) baculum with an under-developed proximal process (mean score = 1.8). Arrow indicates the proximal process. Scale = 1cm.

Mink were killed as humanely as possible. Killing methods followed improved guidelines from the University of Guelph and Michigan State University. Since CO_2_ is highly aversive to mink [102], we anaesthetised the animals first. Each mink was placed individually in a transparent plastic 50 l container, into which 10 ml of isoflurane was sprayed. This rendered animals unconscious in less than 60 s with no obvious signs of stress or suffering. The container was then filled with CO_2_ for three minutes to ensure death. Each was then weighed and measured, and certain structures removed. The following organs were weighed fresh to the nearest 0.0001 g, after careful fat removal: brains (cut at the caudal end of the medulla, adrenals, testes, thymus and spleen. To assess fluctuating asymmetry (FA), mandibles were also removed. These were selected as the bones to measure due to their established anatomical landmarks ([103],[104]; Fig. 3); their known vulnerability to stress [105]; and to avoid potential confounds, since development and FA in other skeletal bones could be influenced by stereotypic behaviours. After manual cleaning, morphometrical analyses of the mandibles' labial sides followed those of Galicia Paredes [106]. Landmarks were located using TpsDig software (v. 2.0, [107]; Fig. 3). They were processed by side rather than by individual (i.e. all left mandibles were processed first, then all right mandibles), to ensure that the second set of landmarks for each individual was placed blind to the location of the first set. The presence of FA was verified, before quantifying the asymmetry between sides for each individual [108]: to do this, mandibular FA was calculated using Procrustes analyses to generate a landmark-based index (“FA18”, [108]) which quantified the shape differences between sides. Finally, the last post mortem data came from the *os penis* (baculum). This was removed from each male, and scored for degree of development, since this bone's proximate knob (Fig. 4) grows with age in mink [109] and least weasels (*Mustela nivalis*) [110], and in the latter species its development is testosterone-dependent [110]. To do this, five observers, each blind to mink treatment and previously trained using published images of young *vs*. mature mink bacula [109], independently ranked our 32 bones from the least developed (i.e. a score of 1) to the most developed (a maximum possible score of 32; ties were allowed). Spearman correlations confirmed concordance between the observers; an average rank was then calculated for each male.

### Statistical analyses

Most analyses involved General Linear Models (GLMs, Minitab v. 14: [111]). Data were transformed where necessary; models contained all possible interactions to avoid pooling error terms [112]; alpha was set at 0.05; and results are 1-tailed due to clear *a priori* predictions [113],[114]. GLMs investigating treatment effects on reproduction contained Male and Female treatment (E *vs*. NE) as independent variables, along with Year, and Male pair (a random effect) nested in Female Treatment. Logistic regressions were conducted for paternity, since this was a categorical variable.

We investigated first how treatment affected phenotypes in the relevant sex, and second, which of these specific housing-influenced aspect(s) of phenotype predicted any observed reproductive benefit. In practice enrichment only affected male reproduction. We therefore ran GLMs that used Male copulation number as the dependent variable, and each housing-influenced aspect of phenotype as an independent variable, blocking for Female treatment, Year, and Male pair nested in Female treatment (‘Analysis 1’). For each variable that these tests identified as significant, we ran a second GLM (‘Analysis 2’), using the proportion of each female's total number of copulations allocated to her E mating partner as the dependent variable; and the absolute difference in the trait of interest's magnitude between that female's E male and his NE rival as the independent variable. Analyses 1 and 2 involved continuous dependent variables that proved to be non-orthogonal [115]; we therefore used sequential sums of squares to calculate F ratios, placing the trait of interest as the last main effect after the blocking factors [116]. These GLMs investigating underlying mechanisms generated 21 P values and we therefore used “false discovery rate” procedures (FDR; [117]) to reduce risks of Type I error. Previously significant results that did not pass these more stringent alphas are subsequently reported as trends.

## Results

### Copulation number: effects of individual and housing, and implications for paternity

Housing conditions did not significantly influence female mating behaviour (see Table 1), nor female chances to produce a litter (odds ratio of non-enriched:enriched = 1.67, Z = 0.57, p = 0.283), nor litter size, nor offspring attributes - although only 17 females whelped and only 10 had kits surviving past PND 4. Male treatment, however, affected the numbers of copulations obtained: NE males were less successful (F_1,14_ = 3.20, p = 0.047; see Table 1). Individual males' copulation numbers also covaried across the two test years (F_1,28_ = 7.48, p = 0.011), showing that it was, or reflected, a stable male trait.

**Table 1:** Effects of environmental enrichment on reproductive variables (see text for effects of enrichment on likelihood of producing progeny).

		Males				Females			
		*Mean*	*SE*	*Statistic*	*p*	*Mean*	*SE*	*Statistic*	*p*
**No. of copulations**	E	**3.25**	**0.56**	**F_1,14_ = 3.20**	**p = 0.047**	1.97	0.43	F_1,14_ = 2.27	p = 0.154
	NE	**1.91**	**0.37**			3.19	0.52		
**Litter size**	E	2.25	0.92	F_1,13_ = 0.47	p = 0.505	2.83	0.56	F_1,13_ = 0.32	p = 0.580
	NE	2.92	0.52			2.33	0.48		
**Average kit weight at birth (g)**	E	11.53	1.481	F_1,13_ = 1.56	p = 0.234	13.99	1.600	F_1,13_ = 0.69	p = 0.210
	NE	14.29	1.481			11.83	1.352		

Significant effects are in bold. Means are raw means.

Paternity analyses revealed that all the kits per litter were sired by just one male. Male treatment did not significantly predict likelihood of paternity (odds ratio of non-enriched:enriched = 1, Z = −0.00, p = 0.500); however, exploratory comparisons of paternity data with behaviour showed that in Year 2 (but not Year 1, p>0.10 in all analyses), mating more often predicted a higher probability of paternity (F_1,10_ = 8.82, p = 0.014).

### Which aspects of male phenotype were affected by enrichment?

Housing-type affected male behaviour, physiology and anatomy (Table 2). NE males showed evidence of increased HPA activity and its predicted effects on reproductive physiology: concentrations of excreted faecal metabolites were higher for glucocorticoids, and lower for testosterone and epiandrosterone. Bacular proximate knobs were also less developed (although not significantly so after FDR corrections, see Table 2). Furthermore, there was evidence of phenotypic changes that could affect attractiveness or social competence: NE males were lighter, had smaller spleens suggesting poorer cell-mediated immunity, and performed more stereotypy; they were also less symmetrical, although not significantly so after FDR corrections (see Table 2).

**Table 2:** Effects of environmental enrichment on male phenotype.

	E		NE			
	*Mean*	*SE*	*Mean*	*SE*	*Statistic*	*p*
**Adrenal weight (g)** *	0.1311	0.00698	0.1283	0.00676	F_1,29_ = 0.08	p = 0.387
**Fecal glucocorticoid metabolites (ng/g)**	**185**	**42.5**	**299**	**41.6**	**F_1,29_ = 8.33**	**p = 0.003**
**Testis weight (g)** *	6.769	0.2656	6.393	0.2656	F_1,30_ = 1.02	p = 0.160
**Testosterone (ng/g)** §	**60.01**	**4.376**	**33.79**	**4.220**	**F_1,28_ = 16.53**	**p<0.001**
**Epiandrosterone (µ/g)**	**17.083**	**1.953**	**5.809**	**1.883**	**F_1,28_ = 15.34**	**p = 0.001**
**Baculum proximal process development score**	**18.61**	**1.778**	**12.64**	**1.721**	**F_1,30_ = 5.37**	**p = 0.014**
**Thymus weight (g)** *	1.156	0.1251	1.102	0.1211	F_1,29_ = 0.09	p = 0.380
**Spleen weight (g)** * ¶	**6.457**	**0.4160**	**4.339**	**0.4160**	**F_1,28_ = 12.95**	**p = 0.001**
**Body weight (kg)**	**2.567**	**0.07625**	**2.218**	**0.07625**	**F_1,28_ = 10.51**	**p = 0.003**
**Fluctuating asymmetry**	**0.03589**	**0.00285**	**0.04331**	**0.00295**	**F_1,29_ = 3.27**	**p = 0.040**
**Locomotory stereotypies**	**2.510**	**0.756**	**33.19**	**7.15**	**F_1,30_ = 13.63**	**p = 0.0005**
**Brain weight (g)** *	10.63	0.1938	10.33	0.1877	F_1,28_ = 0.266	p = 0.133

* corrected for body length

§ corrected for levels of fecal glucocorticoid metabolites

¶ corrected for body weight

Significant results are in bold. Means are raw means. High baculum development scores indicate a higher degree of development; high fluctuating asymmetry scores indicate a higher degree of asymmetry.

### Which enrichment effects on male phenotype predicted copulation number?

**Figure 5:**
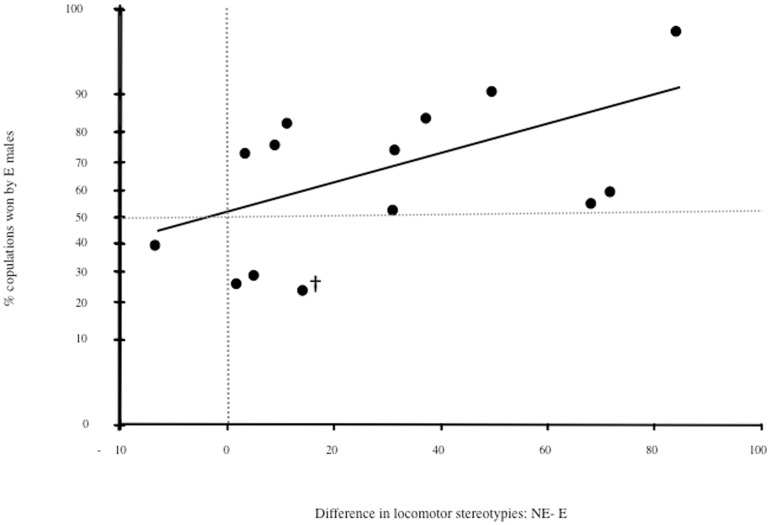
Relationship between locomotory stereotypies and number of copulations. Graph from Analysis 2. “†” indicates the one pair in which the NE male stereotyped (during two trials).

Stereotypies significantly predicted copulation number (Fig. 5), with the most stereotypic males gaining the fewest copulations (Analysis 1: F_1,29_ = 7.60, p = 0.004; Analysis 2: F_1,24_ = 4.25, p = 0.029). Bodyweights also positively co-varied with copulation number in Year 2 (bodyweight data in Year 1 were not available) - although not significantly after FDR corrections (see Table 3). No other aspect of enrichment-altered male physiology or anatomy covaried with copulation number.

**Table 3:** Relationships between enrichment-affected male traits and male mating success.

Traits	Statistical method	Copulation number
Stereotypic behaviour	Analysis 1	**F_1,29_ = 7.60, p = 0.004 -**
*(Year 1 and Year 2 averaged)*	Analysis 2	**F_1,24_ = 4.25, p = 0.029 -**
Body weight	Analysis 1	**F_1,14_ = 3.46, p = 0.042 +**
	Analysis 2	**F_1,10_ = 6.87, p = 0.013 +**
Baculum development	Analysis 1	F_1,14_ = 1.76, p = 0.103
Glucocorticoid metabolites	Analysis 1	F_1,13_ = 1.21, p = 0.146
Testosterone	Analysis 1	F_1,12_ = 0.14, p = 0.710
Epiandrosterone	Analysis 1	F_1,12_ = 0.82, p = 0.383
Spleen weight	Analysis 1	F_1,12_ = 0.00, p = 0.981

Significant results are in bold. -  =  negative relationship; +  =  positive relationship.

## Discussion

Our results for male copulatory behaviour supported the long-standing, previously untested hypothesis that barren environments compromise breeding in captive carnivores. The poorer performance of non-enriched male mink compared to enriched conspecifics indicated weaker libido [118] and/or reduced attractiveness to females [119]: both problematic in the males of some captive-bred wild species [1],[2],[3],[4],[5],[6],[120]. Since multiple matings increase males' chances of fertilization in some species [4],[121], this result also suggests that barren environments could reduce abilities to father offspring. We found no direct evidence for this, but some indirect support: in Year 2, males that successfully sired litters also copulated more. Future work should now investigate this further, and also assess other potential benefits of enriching males, including improved sperm motility [27], male libido [122], attractiveness to females, and enhanced maternal investment in their offspring, by the dams mated to them [40],[123].

Housing conditions did not apparently affect female receptivity, in contrast. Housing conditions also did not seem to affect female fertility (although statistical power was low here because few females reproduced, likely reflecting deficits in the indoor lighting's brightness or spectral range [124]). This aspect of our work therefore requires replication, since the apparent absence of effects on females could be attributed to Type II error, especially given that environmental enrichment increases the number of infants successfully weaned by females in farmed mink [79]. However, it is also possible that male reproductive success is genuinely more sensitive to the physical and sensory properties of their rearing and housing conditions. As evidence, over 95% of female mink successfully conceive litters on commercial fur farms [125], suggesting that their reproduction is rather resilient to barren housing conditions; whereas the same is not true for males, since over 10% do not mate (G. Mason, unpublished data). Furthermore, in non-enriched enclosures, breeding also appears to be more problematic for males than females in European mink, giant pandas and black-footed ferrets [1],[4],[126]; while likewise in flies, barren rearing environments decrease the copulations gained by males but not by females [74],[75].

We then investigated three non-mutually exclusive potential mediators that could explain why non-enriched males are handicapped in mate choice competitions. One widely-suggested problem for NE housed animals is that increased HPA activity will have knock-on detrimental effects on the HPG endocrine axis. Consistent with this, faecal metabolites revealed barren environments to elevate circulating concentrations of cortisol, and perhaps reduce androgen levels (the lack of fully-validated androgen assays for mink necessitating caution here). The bacula of non-enriched males also tended to have less well-developed proximal processes, a result that now needs replication. The second set of mediators investigated were enrichment-induced changes in male morphology or physiology that are likely to affect attractiveness. Mate choice research provides considerable evidence that developmental stress can create stunted, asymmetrical, immune-compromised phenotypes that females generally prefer not to mate with, yet these adverse implications of poor rearing environments are never considered by captive breeding programmes. Consistent with this hypothesis, non-enriched males tended to be more asymmetrical (significant until FDR corrections). They also had smaller spleens, suggesting that immunological benefits of enrichment should now be investigated in depth (e.g. via the histological examination of splenic germinal centre numbers and structure [127]). Non-enriched males were also lighter: an effect whose possible role in their poorer copulatory performance now needs replicating.

Stereotypy was at the heart of our third hypothesis: that barren environments make animals less socially competent by reducing intelligence and/or behavioural flexibility. Even though barren rearing conditions cause well-understood learning, memory and forebrain function deficits [57],[59], the potential implications of such effects for courtship, copulation, maternal care and other intra-specific interactions have to date not been studied. We did not find the brain weight effects reported for rodents, but such data are prone to measurement error. Future studies should therefore assess subjects' learning abilities, and also section brains for histology, in order to measure cortical thickness and hippocampal volume (e.g. [57]). However, we did find strong evidence that captivity-induced basal ganglia changes have adverse socio-sexual implications: non-enriched males who, prior to the mating season, spent a relatively large proportion of their home cage active time budget repetitively pacing, nodding or head-twirling, went on to gain little mating success during mate choice trials. This negative impact of stereotypy on copulation number was not because highly stereotypic males performed stereotypies during the tests themselves (with bouts then directly interfering with sexual behaviour): stereotypy was only observed twice (in just one male) in all 504 mating trials. Instead, we suspect that subtle behavioural correlates of stereotypy are the key. As outlined in the Introduction, humans suffering from psychiatric or developmental disorders characterised by stereotypic behaviour (e.g. schizophrenia; autism) often have problematic or no sexual relationships [128],[129],[130] because they are socially odd [131]: behaviourally and conversationally inflexible, and poor at responding appropriately to others' actions, words or facial expressions. Furthermore, perseveration (inappropriate response repetition) is elevated, not just in these stereotypic humans, but in all stereotypic captive animal species tested to date - including two other Carnivora [132],[133] as well as mink [71],[83]. We therefore suspect that non-enriched, highly stereotypic male mink are inappropiately repetitive or inflexible during courtship, perhaps in their courtship vocalizations (‘chuckling’ [134]) or play-like interactions with receptive females: a hypothesis that now needs to be tested directly.

Small, non-enriched cages are still common in breeding centres for European mink, black-footed ferrets and other endangered carnivores [1],[3],[84],[135],[136]. Currently, breeding problems in these and other captive wild animals are often addressed with reproductive medicine or technology (e.g. artificial insemination [2],[137]). Our results confirm what has been long-suspected (e.g. [4],[137]): that enriching rearing conditions could provide an alternative solution (especially for males). This solution has the added ethical benefit of enhancing animal welfare. We suspect that our results may even have implications beyond captive wild carnivores, suggesting needs for additional research. Suboptimal housing has been suggested to cause breeding problems in other wild taxa (e.g. black rhinoceroses *Diceros bicornis* and white rhinoceroses *Ceratotherium simum*, [138]). Furthermore, stereotypic behaviours are extremely prevalent in other captive populations (including laboratory and farmed animals), being performed by tens or even hundreds of millions of individuals [24]. Additionally, small impoverished enclosures remain common for wild animals [139], and standard for billions of farm and research animals [139]. These are likely to affect brain development even in species that tend not to stereotype (e.g. rats, [140]). We therefore suspect that the perseveration of stereotypic individuals, as well as the learning impairments likely in captive-bred animals, may prove to have widespread socio-sexual consequences across diverse captive species, especially for complex courtship (e.g. duetting), maternal care, and other interactions with conspecifics that require responsiveness and flexibility.
